# Traditional Chinese Medicine Regulating Lymphangiogenesis: A Literature Review

**DOI:** 10.3389/fphar.2020.01259

**Published:** 2020-09-03

**Authors:** Longping Peng, Yidan Dong, Hua Fan, Min Cao, Qiong Wu, Yi Wang, Chang Zhou, Shuchun Li, Cheng Zhao, Youhua Wang

**Affiliations:** ^1^ Cardiovascular Department, Longhua Hospital, Shanghai University of Traditional Chinese Medicine, Shanghai, China; ^2^ Vascular Disease Department, Shanghai Traditional Chinese Medicine Integrated Hospital, Shanghai University of Traditional Chinese Medicine, Shanghai, China

**Keywords:** lymphangiogenesis, traditional Chinese medicine, inhibitory factor, facilitating factor, promoting factor

## Abstract

Lymphatic vessels, as an important part of the lymphatic system, form a fine vascular system in humans and play an important role in regulating fluid homeostasis, assisting immune surveillance and transporting dietary lipids. Dysfunction of lymphatic vessels can cause many diseases, including cancer, cardiovascular diseases, lymphedema, inflammation, rheumatoid arthritis. Research on lymphangiogenesis has become increasingly important over the last few decades. Nevertheless, the explicit role of regulating lymphangiogenesis in preventing and treating diseases remains unclear owing to the lack of a deeper understanding of the cellular and molecular pathways of the specific and tissue-specific changes in lymphangiopathy. TCM, consisting of compound extracted from TCM, Injections of single TCM and formula, is an important complementary strategy for treating disease in China. Lots of valuable traditional Chinese medicines are used as substitutes or supplements in western countries. As one of the main natural resources, these TCM are widely used in new drug research and development in Asia. Moreover, as a historical and cultural heritage, TCM has been widely applied to clinical research on lymphangiogenesis leveraging new technologies recently. Available studies show that TCM has an explicit effect on the regulation of lymphatic regeneration. This review aims to clarify the function and mechanisms, especially the inhibitory effect of TCM in facilitating and inhibiting lymphatic regeneration.

## Introduction

The lymphatic vasculature, the precollectors and collecting vessels, absorbed interstitial fluid, and solutes from the extracellular space, is composed of monolayer overlapping LECs, which are derived mainly from the vascular endothelial cells in the process of embryonic development (Vaahtomeri et al., 2017). Lymphangiogenesis is the production of the new lymphatic vasculature on the basis of initial lymphatic vessel (Stacker et al., 2014). Dysfunction of lymphatic vessels can cause many diseases, including cancer, cardiovascular diseases, lymphedema, inflammation, rheumatoid arthritis (Kunstfeld et al., 2004; Alitalo et al., 2005; Kajiya and Detmar, 2006; Polzer et al., 2008; Aspelund et al., 2016). For example, numerous works on xenogeneic tumor transplantation models in mice have demonstrated that targeting the VEGFC-VEGFR3 and VEGFD-VEGFR3 axis can inhibit tumor lymphangiogenesis, lymphadenopathy, and lymph node metastasis (Stacker et al., 2001; Karpanen et al., 2001; He et al., 2002; Achen et al., 2005; He et al., 2005; Lin et al., 2005; Alitalo, 2011). Tumor lymphangiogenesis may be a useful prognostic indicator for human melanoma (35138) and other human tumor lymph node metastasis, which is consistent with the concept that clinically targeted tumor lymphangiogenesis may limit lymph node metastasis and potential distant organ metastasis (Dadras et al., 2003; Dadras et al., 2005; Stacker et al., 2014). But so far, most research of therapeutic lymphangiogenesis focused on the delivery of the VEGFC gene or the VEGFC protein, which stimulates lymphangiogenesis in its pro-teolytically processed mature form (Joukov et al., 1997; Jha et al., 2017). As early as 40 years ago, cardiac lymph function has been confirmed to alter in cardiovascular disease (Bradham and Parker, 1973). However, only in the past few years, the first published research on the occurrence and role of cardiac lymphatic remodeling in cardiovascular diseases, including myocardial infarction (MI) and chronic heart failure (HF) (Ishikawa et al., 2007; Cui, 2010; Kholová et al., 2011; Klotz et al., 2015; Aspelund et al., 2016; Henri et al., 2016; Dashkevich et al., 2016; Randolph et al., 2017). Early clinical trials of cardiac lymphatics were stopped due to catheter problems, and no data are available on the potential effects on cardiac lymphatics, myocardial edema, or myocardial fibrosis in these patients (Brakenhielm and Alitalo, 2019).

In this review, we aim to provide researchers with the latest advances in the regulation of lymphangiogenesis and the effects and mechanisms of TCM in regulating lymphangiogenesis. TCM, an essential part of traditional culture in China, has age-old history of thousands of years, with both a unique theoretical system and rich clinical experience. In other countries, TCM is often treated as an alternative or complementary medicine, due to a lack of quantitative and objective evaluation criteria. However, TCM has gained growing popularity in many developed countries, such as the United States and Australia. Significantly, some patients prefer to reduce the use of Western medicine suggested by current medical guidelines when the TCM can control the disease (Guo et al., 2013). Available research has demonstrated that TCM can affect lymphangiogenesis in the treatment of different diseases such as cancer (Dai et al., 2017), inflammatory diseases (Liang et al., 2016; Li et al., 2016), rheumatoid arthritis (Chen et al., 2016), lymphedema (Wang et al., 2017). Although TCM plays a role in regulating lymphangiogenesis, their molecular targets are largely unknown. Finding their binding targets would help uncover the mechanisms by which TCM modulates lymphangiogenesis. Much of the research in the growth of lymphatic vessels in the past decades have verified the regulatory effect of TCM. Work on the mechanisms and effects of TCM is still at an early stage. However, all relevant publications indicate that TCM’s potential to regulate lymphangiogenesis deserves further study.

## An Overview of Relevant TCM

Current research indicates that TCM has certain regulatory effects on lymphangiogenesis during disease pathology, which is helpful for the treatment of diseases. TCM is one of the oldest treatments, mainly comprising natural medication (also called Chinese herbal medicine), physiotherapy (e.g., TuiNa, QiGong), acupuncture (Tang et al., 2008). Chinese herbal medicine—an under developed biological resource—is important for drug discovery and development (Li and Vederas, 2009). As an example, DHA, artemether and artesunate, the derivative of artemisinin, has used clinically for the treatment of cerebral malaria and falciparum malaria with good curative effect (Arinaitwe et al., 2009; Lisewski et al., 2014; Wang et al., 2015).

**Figure 1:**
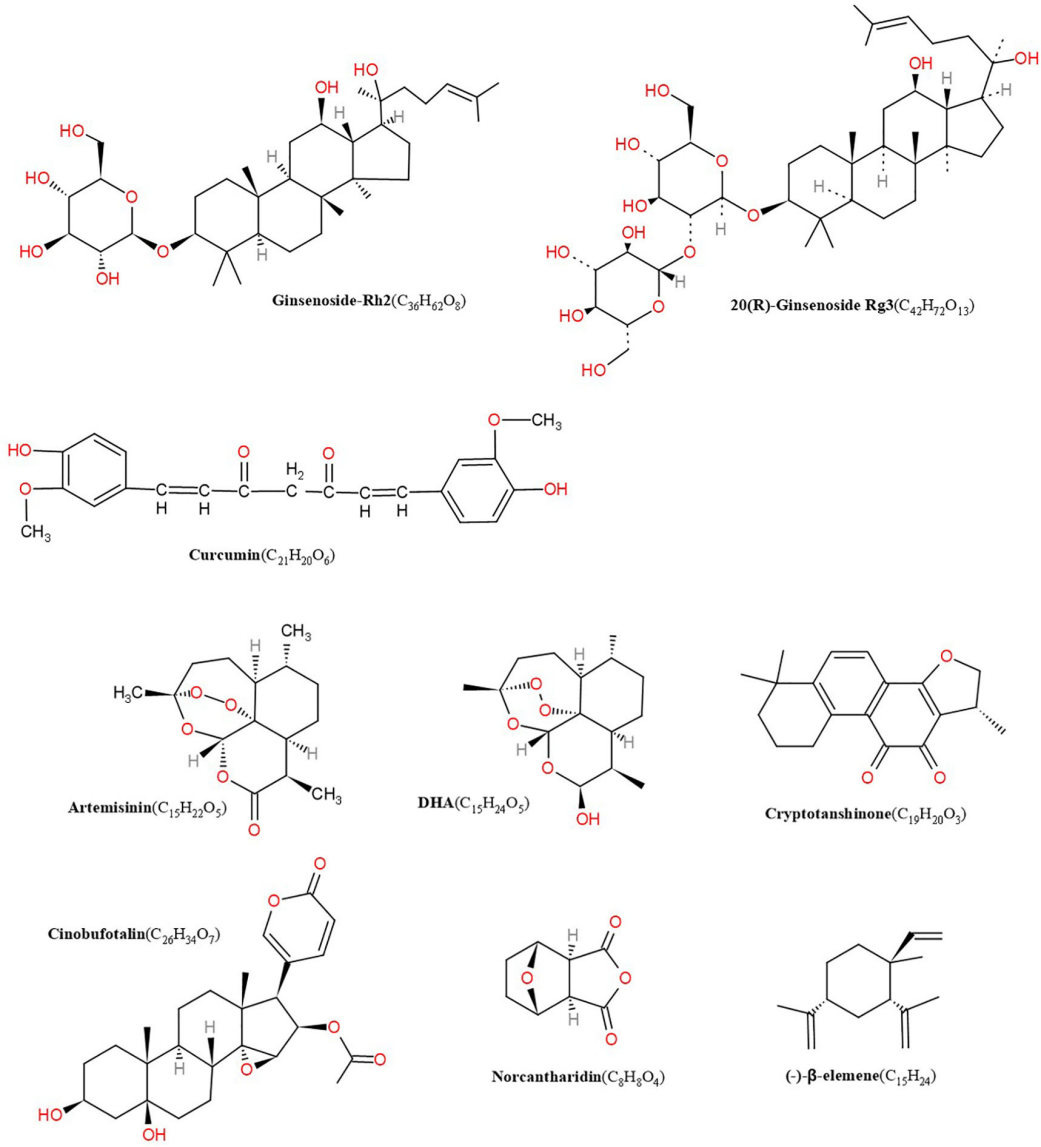
Structures of artemisinin, DHA, cryptotanshinone, cinobufotalin, norcantharidin, β-elemene, GS-Rh2, ginsenoside Rg3 (GS-Rg3), curcumin.

Chinese herbal medicine covers single chinese herbal, effective extraction ingredients of single TCM and Chinese medicine formulas. Available evidence suggest that some TCM extractions, such as ART (Wang J. et al., 2008), DHA (Wang et al., 2007), GS-Rg3 (Li et al., 2009; Dai et al., 2014), GS-Rh2 (Wang Q et al., 2008), CPT (Luo et al., 2011), β-elemene (Yan et al., 2014), curcumin (Wang et al., 2015), and norcantharidin (Liu et al., 2012), have a potent lymphangiogenesis regulatory effect and may alter the disease course. Here, we provide the structure of related compounds from single TCMs that regulate lymphangiogenesis (**Figure 1**).

It is an important part of the TCM theory, and an important embodiment of TCM theory used in clinical practice. In the current clinical treatment, the application of Chinese herb recipe is extensive, and it still has more obvious advantages than single Chinese herbal or the monomer from Chinese herbal. Interestingly, studies show that some TCM formulas can effectively regulate lymphangiogenesis.

Currently, except for a few studies involving HIF-1, Ang-1/2, and MMP-9. Others are mainly focused on the effects of TCM in regulating lymphangiogenesis in cancer, and the mechanistic research focuses on VEGF/LECs. In the pathological progress of various diseases, such as lymphedema, cardiovascular diseases, and atherosclerosis, there is still large space to explore the effects and mechanisms of TCM on lymphangiogenesis.

## Regulatory Factors of Lymphangiogenesis

The lymphatic vessels are crucial for assisting immune function, regulating tissue fluid homeostasis, and accelerating the absorption of dietary fats. The growth of lymphatic vessels occur during wound healing, embryonic development, and under pathological conditions of different diseases, including cardiovascular diseases, cancer, obesity, hypertension, inflammatory diseases, lymphedema, and atherosclerosis. Nevertheless, the molecular mechanisms regulating the growth of lymphatic vessels are far less understood. Certainly, our knowledge of the effects and regulatory mechanisms of the lymphatic vascular system will give rise to creative and crucial insights in biology and clinical medicine. Lymphangiogenesis, the growth of lymphatic vessels, has captured growing attention in the past few decades, mainly thanks to advances in the discovery of regulatory factors and markers particular to the lymphatic endothelium. This promoted the research on regulation of lymphangiogenesis by TCM.

Much of the research has demonstrated that the development of lymphangiogenic factors, lymphangiogenesis, and the remodel of lymphatic vessels can associate with some progression of the disease. For example, Lymphatic vessels modulate the immune response of cancer and restrict metastasis, providing targets for cancer treatment. Importantly, lymphangiogenesis exists in tissues not only during the metastasis of the tumor but also in inflammation and wound healing. Consequently, the organization of Lymphatic factors makes it possible to target lymphatic vessels in the disease. Then, we summarize the latest progress that has exceedingly advanced our understanding of the factors and mechanisms of lymphangiogenesis, including promoters (**Table 1** and inhibitors **Table 2**).

**Table 1:** Lymphangiogenesis (LY) facilitating factors with the function.

Facilitating factor of LY	Mechanism of action (MOA)	References
**VEGF**	Binding specific receptors to stimulate lymphangiogenesis	(Kunstfeld et al., 2004).
	VEGF-A binds to VEGFR-1/fms-like tyrosine kinase 1 (FLT-1)	(Joukov et al., 1996).
	VEGFR-2/human kinase insert domain receptor (KDR)	(Achen et al., 1998).
	VEGF-C and VEGF-D bind to VEGFR-3/FLT4, and upon proteolytic processing can bind to VEGFR-2	(Jeltsch et al., 1997).
	Activating intracellular signaling pathways such as map kinases Erk1/2 and Akt	(Veikkola et al., 2001)
**FGF-2**	Activating FGFR-1, induce lymphangiogenesis direatly	(Kubo et al., 2002). (Chang et al., 2004).
	Facilitating the increase of VEGF-C/D and stimulate lymphangiogenesis	(Cao et al., 2012). (Kazenwadel et al., 2012).
**IGF-1/IGF-2**	Combined with IGFR to induce lymphangiogenesis directly	(Björndahl et al., 2005). (Li et al., 2013).
	Through other growth factor receptor systems induces lymphangiogenesis indirectly	(Morgillo et al., 2013)
**PDGF-BB**	Binding to PDGF-BB receptor to induces lymphatic vessel growth directly	(Eriksson et al., 2003). (Cao et al., 2004).
	Activating intracellular signaling pathways such as map kinases Erk1/2 and Akt	(Schoppmann et al., 2013)
**Angopoietin (Ang)**	Ang-1 induces Tie-2 phosphorylation in LEC and promotes lymphatic budding and growth	(D’Amico et al., 2014).
	Ang-2,Ang-3 and Ang-4 Promotes lymphangiogenesis in wound healing models, with the specific mechanism is not clear	(Tammela et al., 2005). (Morisada et al., 2005). (Kim et al., 2007)
**HGF**	Binding to c-Met to increase expression of podoplanin and LYVE-1, induces lymphatic vessel growth directly	(Saito et al., 2006). (Jiang et al., 1999).
** **	Through VEGFR-3 promotes lymphangiogenesis Indirectly	(Cao et al., 2006). (Zhang et al., 2015)
**TNF**	TNF-α/LT-α, up-regulates the protein expression of VEGF-D through the ERK1/2-AP1 pathway, and upregulates VEGF-C expression through macrophages	(Mohamadzadeh et al., 1998). (Baluk et al., 2005). (Hong et al., 2016). (Hjelmström et al., 2000). (Mounzer et al., 2010).
** **	LT-α, through LTα3 signaling of the TNFR1 receptor to induce lymphangiogenesis	(Cuff et al., 1998). (Ristimäki et al., 1998). (Sacca et al., 1998). (Furtado et al., 2007)
**IL**	IL-1β, increases the expression of VEGF-C/D in macrophages	(Baluk et al., 2013).
** **	IL-33, through the ST2/TRAF6-mediated Akt/eNOS/NO signaling pathway	(Han et al., 2017)
**Adenosine**	Stimulating lymphangiogenesis through the involvement of macrophages	(Lenoir et al., 2014)
**Netrin-4**	Activating small GTPases and Src family kinases/FAK, and down-regulating tight junction proteins	(Sleeman and Thiele, 2009).
** **	Activating p42/p44 MAPkinase, Akt/PI3kinase and mTor signaling pathways	(Larrieu-Lahargue et al., 2010)
**CLEC14A**	Interacting with VEGF-3 to regulate the expression and Signal Transduction of VEGFR-2 and VEGFR-3	(Lee et al., 2017)
**Adrenomedullin**	Activating Notch signaling pathway and transactivating VEGFR-2	(Nicoli et al., 2008). (Yurugi-Kobayashi et al., 2006). (Guidolin et al., 2008)
**ET-1**	Binding to ETBR, stimulating the expression of HIF-1a, up-regulating the expression of VEGF-C, VEGFR-3 and VEGF-A	(Spinella et al., 2009). (Irigoyen et al., 2007). (Ota et al., 2007). (Schoppmann et al., 2006). (Katsuta et al., 2005).

**Table 2:** Lymphangiogenesis inhibitory factors with the functional mechanisms.

Inhibitory factor of LY	Mechanism of action (MOA)	Ref
**TSP-1**	Linked with CD36 in macrophages to reduce the expression levels of VEGF-C and VEGF-D	(Cursiefen et al., 2011).
**TGF-β1**	TGF-β1,reducing the recruitment, proliferation, and tubule formation of LEC	(Oka et al., 2008). (Clavin et al., 2008). (Avraham et al., 2010).
**IFN-γ**	Through serial events of signaling by IFN-γ *via* IFNGR-1 and/or IFNGR-2, JAK, and STAT-1 phosphorylation, causes the downregulation of Prox-1, LYVE-1, and podoplanin in LECs	(Kataru et al., 2011).
**BMP2**	Strongly inhibits the expression of PROX1, the master regulator for the lymphatic fate, in a SMAD-dependent and miRNA-dependent manner	(Dunworth et al., 2014).
**Endostatin**	Decreasing the VEGF-C levels	(Brideau et al., 2007).

## TCM for Facilitating Lymphangiogenesis (**Table 3**)

Compared with the lymphangiogenesis inhibition of TCM, the research on promoting lymphangiogenesis by TCM is relatively limited until now. Only one TCM monomer and two formulas work.

**Table 3:** TCM for facilitating lymphangiogenesis.

Drug	Model	Dosage and Route of administration	Mechanism	The herbal composition (including the scientific name)	Ref
GS-Rg1	Male Spraguee Dawley (SD) rats	10mg/kg/day Gavage administration	Enhancing the expression levels of VEGF-C and VEGFR-3	Extracted from panax –ginseng (*Panax ginseng C.A.Mey.*)	(Yu et al., 2016)
PNS	1) The transgenic zebrafish line (fli1:egfp; gata1:dsred) 2) A murine LEC cell line established from Freund’s adjuvant-induced benign lymphangiomas	10, 50, 100μM Immersion administration	Up-regulating VEGF-C expression and activation of ERK1/2, PI3K and P38MAPK signaling	Extracted from Panaxnotoginseng (*Panax notoginseng (Burkill) F. H. Chen ex C. H.*)	(Li et al., 2016)
DHJST	1) The lines of TNF transgenic mice 2) The transgenic zebrafish line (fli1:egfp; gata1:dsRed)	1) 12 g/kg Gavage administration 2) 10, 30, 100 μg/mL ImmersionAdministration	Not mentioned	Composed of *Radix Angelicae Pubescentis, Herba Taxilli, Radix Acanthopanacis Bidentatae, Herba Asari, Radix Gentianae Macrophyllae, Cortex Cinnamomi, Eucommia, Rhizoma Chuanxiong, Radix Saposhnikoviae, Radix Saposhnikoviae, liquorice, angelica, peony, Rehmannia, Ginseng*, and *poria*	(Chen et al., 2016)
FJHQ	1) The lines of TNF transgenic mice 2) The transgenic zebrafish line (fli1:egfp; gata1:dsRed)	1) 12 g/kg Gavage administration 2) 10, 30, 100 μg/mL Immersion Administration	Not mentioned	Composed of *Stephania tetrandra S.Moore, Astragalus membranaceus (Fisch.) Bunge., Atractylodes macrocephala Koidz. Glycyrrhiza uralensis Fisch*	(Wang et al., 2017)

**Figure 2:**
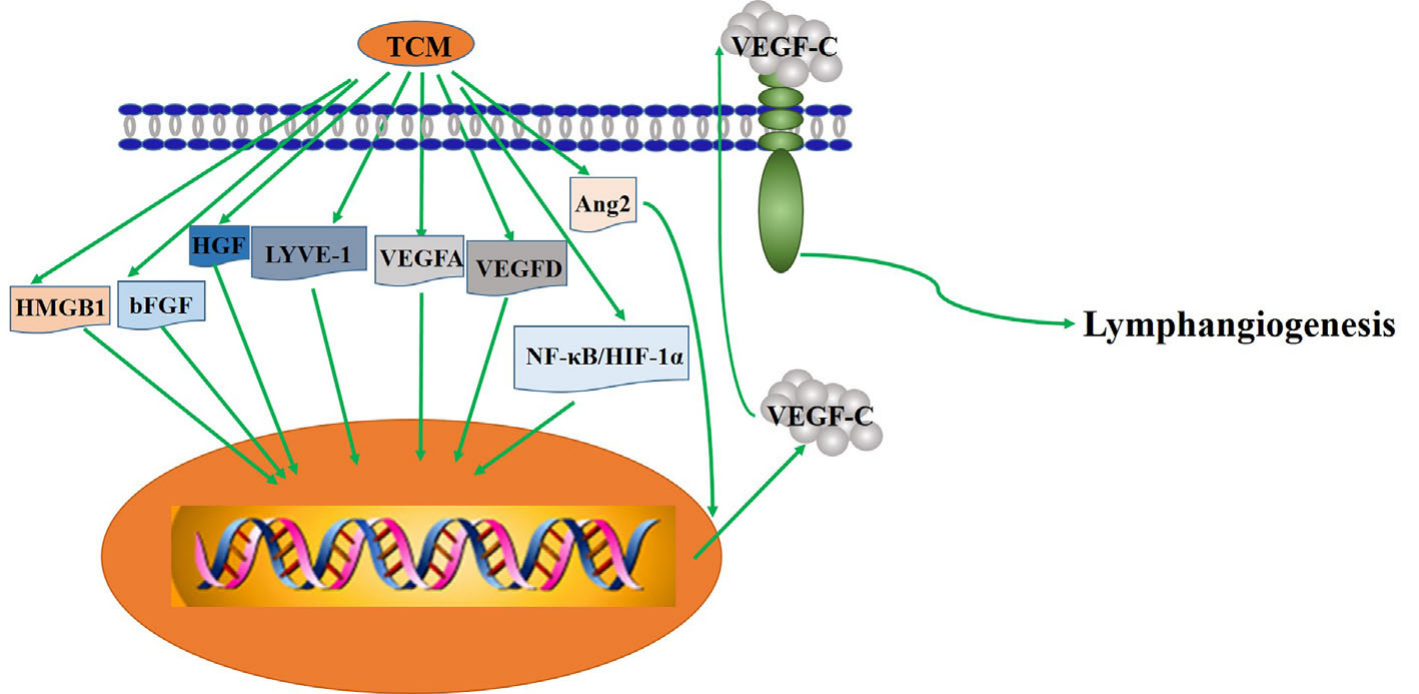
The key mechanism of TCM regulating lymphangiogenesis.

GS-Rg1 is one of the most active ingredients extracted mainly from panax ginseng (**Figure 2**). Yu et al. observed the effect of GS-Rg1 on the lymphatic transport of silica during experimental silicosis. It was found that GS-Rg1 increases the density of pulmonary LVD and facilitates lymphangiogenesis by enhancing the protein and mRNA expression of VEGF-C and VEGFR-3 (Yu et al., 2016).

PNS used as a drug in China for treating cardiovascular and cerebrovascular diseases, are a mixture extracted from Panaxnotoginseng (**Figure 2**). When studied the mechanism and the effect of PNS on the growth of lymphatic vessels, it was found that PNS activates the growth of lymphatic vessels both *in vitro* and *in vivo via* upregulating the expression of VEGF-C and activation of ERK1/2, PI3K, and P38MAPK signaling. These findings suggest that PNS might be a suitable and promising therapeutic agent for the treatment of lymphatic system impairment-related disease (Li et al., 2016).

TCM formula—DHJST, widely used in China to treat rheumatoid arthritis, is a proprietary Chinese medicine. Two animal models, zebrafish and mice, were used to study the effect of DHJST on lymphangiogenesis. The zebrafish research showed that DHJST facilitated the formation of Lymphatic vessels. Furthermore, DHJST promotes lymphangiogenesis and the lymphatic drainage function of mice (Chen et al., 2016). Interestingly, in order to study the mechanism of FJHQ Decoction treats for joint swelling, Wang et al. also used two animal models, mice (TNF-α transgenic mice) and zebrafish. The results show that FJHQ decoction promoted lymphatic drainage and lymphangion genesis to alleviate joint swelling (Wang et al., 2017).

Although the TCM theory of Chinese herb recipe lacks synchronous TCM pathology or pathogenesis research, there are already international successful examples of Chinese herb recipe as a drug for modern medical pharmacology research, and there are efforts to furtherly introduce it into the mainstream of science (Normile, 2003). Chinese herb recipe is worth researching.

## TCM for Inhibiting Lymphangiogenesis

Lymphangiogenic plays an important role in the pathology of disease. For instance, lymphatic factors (like VEGF-A and VEGFC) can bring about tumor metastasis and spread in mouse cancer models (Tobler and Detmar, 2006). In addition, the inhibitory effect of TCM on lymphangiogenesis is being explored *in vivo* and *in vitro* to access its clinical value.

### Extracts of Single TCM (**Table 4**)

ART, extracted from the Chinese herb Artemisia annua L, is a novel effective antimalarial drug. Experimental analyses have revealed that ART can downregulate the level of VEGF-C and reduce lymphangiogenesis in *in vivo* and *in vitro* (Wang J. et al., 2008). Besides, DHA, extracted from Chinese plant artemisinin for the first time, is a semisynthetic agent. DHA dose-dependently inhibited the migration and formation of LECs and upregulated the mRNA level of the proapoptotic gene bax but significantly downregulated the mRNA expression of antiapoptotic bcl-2. What’s more, DHA could apparently attenuate the mRNA and protein expressions of VEGFR-3/Flt-4 (Wang et al., 2007), which suggest that DHA might be a candidate as an anti-lymphangiogenesis inhibitor.

**Table 4:** TCM for inhibiting lymphangiogenesis (extracts of single TCM).

Drug	Model	Dosage and route of administration	Mechanism
ART (Wang J. et al., 2008)	1) Specific pathogen-free female C57BL/6 mice 2) Mouse Lewis lung carcinoma cells	1) 50 mg/kg daily 2 weeks 2) 20μM/24h Oral/Immersion administration	Downregulating the level of VEGF-C
DHA (Wang et al., 2007)	Murine LECs	2.5, 10 μg/mL Immersion administration	1) Inhibiting the migration and formation of LECs 2) Attenuating the mRNA and protein levels of VEGFR-3/Flt-4.
GS-Rh2 (Wang Q. et al., 2008)	Transplanted-tumor mice	20 mg/kg Gavage administration	Down-regulating the expression of the junction adhesion molecul
VRIN (Dai et al., 2014)	Gastric cancer model in nude mice	1 mg/kg 0.1 mg/kg Tail vein injection	Down regulating the expression of VEGF-C
GS- Rg3 (Li et al., 2009)	LECs which ducted from healthy pig	10, 20, 30, 50 μg/mL Immersion administration	Inducing LECs apoptosis
β-Elemene (Yan et al., 2014)	1) Human gastric cancer cells MKN-45 2) Human gastric cancer in nude mice	25, 50, 100 mg/kg Oral administration	Not specific
Gecko (Liu et al., 2008)	1) The transplanted tumor model of the mouse S180 sarcoma 2) Human esophageal carcinoma cells	13.5, 9, 4.5 g/kg Oral administration	Down regulating protein expression of VEGF-C and bFGF
Gecko powder (Zhang et al., 2011)	BALB/c tumor-bearing mice	1.2 g/kg Gavage administration	Affecting the expression of VEGF-C and not specific
GSPP (Ding et al., 2016)	1) Human LECs 2) Nude mice models	1) 10/100 μg/mL Immersion administration 2) 200 mg/kg/d Intraperitoneal injection	Inhibiting the expression of bFGF
Curcumin (Wang et al., 2015)	1) Human dermal microvascular lymphatic endothelial cells 2) C57BL/6mice	3, 10 μM 9 μg Immersion administration	Inhibited expressions of VEGF receptors (VEGFR2 and VEGFR3), as well as downstream signaling such as phosphorylation of ERK and FAK.
Curcumin (Matsuo et al., 2007)	TR-LE cells	20 μM/9 h	Inhibiting the phosphorylation of Akt and enzymatic activity of MMP-2
Curcumin (Da et al., 2019)	The Gastric cancer AGS and SGC-7901 cell lines	0, 10, 15, 20 μg/mL Immersion administration	Eliminated the mRNA and protein expression levels of HMGB1 and VEGF-D.
CPT (Luo et al., 2011)	The murine lymphatic endothelial cells	0–10 µM Immersion administration	Inhibiting LEC tube formation: 1) Inhibiting VEGFR-3-mediated ERK1/2 phosphorylation, 2) Inhibiting expression of the small GTPases.
NCTD (Liu et al., 2012)	Human LECs	1.25–15.00μg/mL Immersion administration	Blocking VEGF-C and VEGF-D/VEGFR-3 *in vitro* simultaneously.
NCTD (Yuan et al., 2015)	Human dermal lymphatic endothelial cells	0, 7.5, 15, 30, 60, 90 nmol/L Immersion administration	Down regulating the expression of VEGF-C and VEGF-D
NCTD (Li et al., 2015)	1) The in-situ colonic xenografts in nude mice 2) Human colonic adenocarcinoma HT-29 cell line	1) 28mg/kg Gavage administration 2) 1.25 – 100 μg/mL Immersion administration	Down regulating VEGF-A, -C, -D/VEGFR-2, -3 signaling pathways
EEHDW (Li et al., 2019)	Human LECs	0, 0.125, 0.25, 0.5 and 1 mg/mL Immersion administration	Down regulation of VEGF−C
Licoricidin (Park et al., 2016)	1) 4T1 mammary tumor tissues in BALB/c mice 2) MCF-10A normal mammary epithelial cells	1) 2 or 4 mg/kg 2) 0–5 μg/mL Immersion administration	Reduced the expression of VEGF-C, VEGF-R3, and LYVE-1
Shikonin (Prangsaengtong et al., 2018)	Human LECs	0.8 µM Immersion administration	1) Interfering the NF-κB/HIF-1α pathway 2) Suppressing VEGF-C and VEGFR-3 mRNA expression.
Wogonin (Kimura and Sumiyoshi, 2013)	1) LM8-bearing mice 2) THP-1 macrophages	1) 25, 50 mg/kg, twice daily 2) 10, 50, 100 μM Oral/Immersion administration	1) Reducing VEGF-C-induced VEGFR-3 phosphorylation 2)Inhibiting the expression of COX-2 and the production IL-1β
Oxyresveratrol (Kimura and Sumiyoshi, 2016)	1) QGY-7701 and SMMC-7721 cells, 2) male mice(lymph node metastasis model)	1) 20, 40, 80μM Immersion administration 2) 20, 40, 60mg/kg Intraperitoneally injected	1) Decreased micro-blood vessel density and micro-lymphatic vessel density, 2) Inhibiting the expressions of CD31, VEGFR3, and VEGF-C
Resveratrol (Liu et al., 2018)	1) M2 macrophage/Human LECs 2) LM8-bearing mice	1) 1,5,10, 25, 50 μM 2) 10, 25 mg/kg, twice daily	1) Inhibited the migration, invasion of VEGF-C-induced Human LECs. 2) Regulatied the activation and differentiation of M2 macrophage 3) Reduced the area of LECs
Hyperforin (Rothley et al., 2009)	1) Female Wistar rats with tumor 2) Primary human LECs, HUVECs	1) 100 μL of 2 mM daily 2 week 2) 5, 30μM 10days	Suppressed the proliferation of LECs
Liposomal honokiol (Wen et al., 2009)	1) Lymph node metastasis models (C57BL/6) mice 2) HUVECs and Human LECs	1) Intraperitoneally injection once a day for 28 days 2) Immersion administration	Inhibiting the VEGFR-3 pathway

In mice with transplanted-tumor *via* inoculating S180 cells under skin, administration of GS-Rh2 inhibited tumor growth and reduced LVD, indicating that the expression of junction adhesion molecule was weakened and that the lymphangiogenesis in xenograft inhibited after treatment with GS-Rh2 (Wang Q. et al., 2008) (**Figure 2**). Dai et al. prepared the VRIN to investigate its effect on the growth and lymphangiogenesis of gastric cancer *in vivo* (**Figure 2**). The results show that VRIN can suppress the growth and lymphatic metastasis of human gastric cancer cell transplantation tumors in nude mice by downregulating VEGF-C expressions (Dai et al., 2014). Using LECs from healthy pig duct, Li et al. found that GS-Rg3 could induce the apoptosis of LECs and evidently inhibit the migration and proliferation of LECs, and the effects were dose-dependent (Li et al., 2009). Noteworthy that the mechanism of GS-Rg3’s effect on LECs needs further study.

VEGF-C and VEGFR-3 are two major regulatory molecules of lymphangiogenesis. β-Elemene could restrain lymphangiogenesis in a dose-dependent pattern by manipulating the VEGF-C and VEGFR-3, which could be regarded as a potential mechanisms for its role in the prevention of lymphatic metastasis (Yan et al., 2014).

By inducing tumor cell apoptosis and downregulating the expression level of VEGF and bFGF, gecko is shown first time to play an anti-tumor role *in vivo* and *in vitro* (Liu et al., 2008). Later, Zhang et al. demonstrated that Gecko lyophilized powder, an active ingredient from the gecko, can inhibit the growth of tumors and also the lymphatic vessels in tumor-bearing mice (**Figure 2**). Combined with previous studies, they speculated that the mechanism of Gecko lyophilized powder on lymphangiogenesis involved affecting the expression of VEGF-C (Zhang et al., 2011). However, a more specific mechanism needs further research. Afterwards, more recent studies by Ding et al. shown that GSPP, an extract of the gecko, acquires the antitumor properties by decreasing the expression of bFGF-inhibiting the growth of lymphatic vessels *in vitro* and *in vivo*, which may further suppress lymphatic metastasis (Ding et al., 2016).

Curcumin is a phenolic pigment extracted from turmeric rhizomes of the ginger family. Curcumin suppressed VEGF-C induced lymphangiogenesis in a Matrigel plug assay in mice, and VEGF-C induced tube formation in human dermal LECs, demonstrating its anti-lymphangiogenic action *in vivo* and *in vitro*. It also demonstrated that curcumin inhibits lymphangiogenesis by inhibiting proliferation, cell cycle progression, and migration of LECs (Wang et al., 2015). Another work further showed that curcumin inhibited the tube-forming process in lymphatic, which partly through suppressing the phosphorylation of Akt and enzymatic activity of MMP-2 (Matsuo et al., 2007). In addition, curcumin significantly inhibited the mRNA andprotein expression of HMGB1 and VEGF-D. It was suggested that curcumin may play an anti-lymphangiogenesis role by inhibiting HMGB1/VEGF-D signaling pathway (Da et al., 2019).

CPT, a natural compound isolated from Danshen (Salvia miltiorrhiza), is a potential antitumor drug. Examined an *in vitro* model (LECs from mouse) pretreated with CPT, Luo et al. suggested that CPT can inhibit the formation of LECs partially by reducing the expression of the small GTPases or partially by suppressing the phosphorylation of ERK1/2 mediated by VEGFR-3 (Luo et al., 2011).

Norcantharidin (NCTD), a demethylated form of cantharidin, is an effective component of cantharidin. In several cell lines and tumor xenograft models, NCTD has been reported to have effective anti-angiogenic and anti-tumor properties (Ho et al., 2001; Fan et al., 2007; Chen et al., 2009; Fan et al., 2010; Deng and Tang, 2011; Zhang et al., 2012). However, its role in tumor associated lymphangiogenesis and lymphatic metastasis is not clear. Liu et al. confirmed for the first time that NCTD can inhibit the proliferation, migration, invasion, and lymphangiogenesis of HLEC; can induce the apoptosis of HLEC in a dose- and time-dependent manner; and can down regulate the protein and/or mRNA expression levels of VEGF-C, VEGF-D, and VEGFR-3 in the lymphangiogenesis of HLEC (Liu et al., 2012). Then, Yuan et al. further demonstrated that NCTD attenuates lymphangiogenesis by down regulating the expression level of VEGF-C and VEGF-D (Yuan et al., 2015). Afterwards, the effect of NCTD on lymphangiogenesis was observed *in vitro* and *in vivo*. It was found that NCTD could directly or indirectly down regulate the expression of VEGF-A, VEGF-C, VEGF-D, VEGFR-2, and VEGFR-3 in the process of lymphangiogenesis *in vitro* and *in vivo* and inhibit lymphangiogenesis (Li et al., 2015).

EEHDW is an ethanol extract of HDW. HDW, the member of the Rubiaceae family of shrubs, tropical herbs, and trees, is a Chinese herb. Li et al. investigated the effects of EEHDW on human LECs model stimulated by VEGF-C. It was found that EEHDW inhibited lymphangiogenesis by down regulating VEGF-C (Li et al., 2019).

Licoricidin, an active compound in the Hexane/Ethanol Extract of Glycyrrhiza Uralensis. Park et al. found that the expression of VEGF-R2, VEGF-C, VEGF-R3, and LYVE-1 decreased in licoricidin treated tumor tissues of mice, indicating that licoricidin can inhibit lymphangiogenesis (Park et al., 2016).

Shikonin, a natural compound found in the root of Lithospermum erythrorhizon, possesses multiple pharmacological activities, such as anti-cancer, anti-inflammation, and anti-angiogenesis properties in *in vitro* and *in vivo* studies (Lee and Magesh, 2008; Lu et al., 2011) (**Figure 2**). It was found that shikonin inhibits pathological lymphangiogenesis, such as tumor induced lymphangiogenesis, by down regulating the expression of VEGF-C and VEGFR-3 genes in lymphangioendothelial cells (Prangsaengtong et al., 2018).

Wogonin, one of the effective components of Scutellaria baicalensis Georgi (Labiatae), is a flavonoid. Studies have shown that wogonin has anti-tumor and anti metastasis effects, which may be related to the inhibition of VEGF-C-induced lymphangiogenesis by inhibiting the expression of COX-2 and the production of IL-1 β in TAMs (Kimura and Sumiyoshi, 2013).

Resveratrol is isolated from Polygonum cuspidatum roots. Works on resveratrol demonstrated that resveratrol-treated condition medium of M2 macrophages can inhibit the migration, invasion of VEGF-C-induced HLEC, and lymphangiogenesis, and resveratrol can reduce the area of LECs in tumors *in vivo*, which suggest that the antitumor effect of resveratrol were partly due to anti-lymphangiogenesis through the regulation of M2 macrophage activation and differentiation (Kimura and Sumiyoshi, 2016).

Oxyresveratrol, trans-2, 4, 3′, 5′-tetrahydroxystilbene, is a derivative of Resveratrol. There is a study demonstrated that Oxyresveratrol significantly anti-tumor effects resulted from suppressing lymphangiogenesis through reducing the expression level of VEGF-C and VEGFR (Liu et al., 2018). Resveratrol and Oxyresveratrol may be a potential therapeutic agent for cancer.

Hyperforin, one of the main active constituents of *Hypericum perforatum* L, is a acylphloroglucinol‐type compound substituted with lipophilic isoprene chains. Aristoforin is new chemical derivatives of hyperforin with improved stability and solubility properties. Both hyperforin and aristoforin significantly suppressed the proliferation of LECs (Rothley et al., 2009).

Honokiol, isolated from the bark of Magnoliae, is a bioactive constituent. Liposomal honokiol exerted a significant inhibitory effect on lymphangiogenesis by downregulation of the level of VEGF‐D in VEGF‐D‐LL/2 cancer cells and interacting neogenesis and formation of LECs directly through the VEGFR‐3 pathway. The findings suggest that liposomal honokiol may be a potential agent against lymphangiogenesis and metastasis (Wen et al., 2009).

### TCM (**Table 5**)

Using LECs from the thoracic duct of pig, Li et al. suggests that Shenmai injection induces the apoptosis of LECs and the inhibition of lymphangiogenesis. Further work is needed to learn more about the mechanism of Shenmai injection on the growth of lymphatic vessels (Li et al., 2011).

**Table 5:** TCM for inhibiting lymphangiogenesis (TCM).

Drug	Model	Dosage and Route of administration	Mechanism	The herbal composition (including the scientific name)	Ref
Shenmai injection	LECs which are ducted from healthy pig	0, 40,80, 120, 160 μg/mL Immersion administration	1) Inhibiting the proliferation of LECs 2) Inducting the apoptosis of LECs	Composed of *Panax ginseng C. A. Mey, Ophiopogon japonicus (Linn. f.) Ker-Gawl.*	(Li et al., 2011)
Astragalus injection	BALB/c nude mice (NIH/3T3^HOOK-RET^ cell)	5mL/kg Intraperitoneal injection	Reducing the expression of MLVD and the positive of rate of HIF-α	*Astragalus membranaceus (Fisch.) Bunge.*	(Li et al., 2014)
Cinobufacini injection	Human LECs	0.105, 0.21, 0.42μg/mL Immersion administration	1) Inhibiting the proliferation, migration and tubular structure formation of HLEC 2) Down regulating the protein expression of VEGFR-3 and HGF	14,15b-Epoxy-3b,5a,16b-trihydroxy-5b,20(22)-bufadienolide 16-acetate	(Gao et al., 2013)

Compared with model group, group pretreatment with *Astragalus* injection, the expressions of MLVD and the positive rate of HIF-1α were significantly reduced in differentiated thyroid cancer (Li et al., 2014).

The cinobufacini injection is a traditional antitumor drug whose mechanism is still unclear (**Figure 2**). The effect of cinobufacini injection on HLEC migration, is through downregulating the expression of HGF and VEGFR-3. Cinobufacini injection can inhibit the migration, proliferation, and tube-like structure formation (Gao et al., 2013).

## TCM Formula (**Table 6**)

### Experimental Research of TCM Formula

Chinese medicine formula plays a significant role in traditional clinical treatment due to its characteristics of targeted treatment. Quite a few studies have found that TCM formulas are also potential inhibitors of lymphangiogenesis.

**Table 6:** TCM for inhibiting lymphangiogenesis (TCM formula).

Drug	Model	Dosage and Route of administration	Mechanism	The herbal composition (including the scientific name)	Ref
ATU I	615 mouse model of Hca-F hepatoma	0.4mL (80g/kg) Hypodermic injection	Down regulating the expression of VEGF-C and Flt-4	Composed of *Glehnia littoralis*, *Asparagus cochinchinensis(Lour)Merr*, *Taxus chinensis (Pilger) Rehd.*, et al.	(Gao et al., 2009)
RKY	Transplantation tumor spontaneous metastasis model	18, 45, 90 g/(kg·d) Gavage administration	Intervening the expressions of VEGF-C and VEGFR3	Composed of *Citrus reticulata Blanco*, *Bupleurum chinense*, *Curcuma zedoaria (Christm.) Rosc.*	(Li and Dang, 2011; Sun and Li, 2013)
JFK	Mice lung cancer model	15, 30, 60 g/kg Oral administration	Lower the number of LYVE-1 positive cells	Composed of *Astragalus membranaceus (Fisch.) Bunge., Glehnia littoralis, Ophiopogon japonicus (Linn. f.) Ker-Gawl., Fructus Ligustri Lucidi, Cornus officinalis Sieb. et Zucc., Gynostemma pentaphyllum (Thunb.) Makino*, et al.	(He et al., 2016; Zhou et al., 2017)
JG-DS	Human LECs	200 μL Immersion administration	Inhibiting the tube formation and migration of HLECs	Composed of *Pinellia ternate, Arisaema erubescens (Wall.) Schott., Wolfiporia cocos, Semen sinapis, Orange peel, Glycyrrhizae.*	(Feng et al., 2016)
PZH	Human CRC cell lines /Human LECs	0, 0.25, 0.5, 0.75 mg/mL Immersion administration	Down regulating the expression of VEGF-C	Composed of *Bos taurus domesticus Gmelin, Moschus, Panaxnotoginseng (Burk.) F.H.Chen, Snake bile.*	(Lin et al., 2016)
ShuGan- JianPi	50 Breast cancer patients	20mL×3/d Oral administration	Decreasing the protein expression of Ang2 Increase the protein expression of nm-23	Composed of *Bupleurum chinense, Citrus reticulata Blanco, Astragalus membranaceus (Fisch.) Bunge., Wolfiporia cocos, Coix lacryma-jobi L.var.mayuen(Roman.) Stapf*	(Jin and Li, 2012)
TaoHong- SiWu	40 Breast cancer patients	200mL×3/d Oral administration	Decreasing the expression of LVD and VEGF-C	Composed of *Prunus persica (L.) Batsch, Carthamus tinctorius L., Angelica sinensis, Ligusticum chuanxiong hort, Rehmannia glutinosa Libosch*, et al.	(Chu et al., 2013)

ATU I is an effective formula for treating advanced cancer, extracted and concentrated from eight kinds of TCM, such as coastal *Glehnia* root, Asparagus, *Scutellaria baicalensis*, etc (**Figure 2**). An inhibitory effect of ATU I on the lymphangiogenesis and the metastasis in liver cancer mice has been verified. The mechanism is associated with the down-regulation of VEGF-C and Flt-4 (Gao et al., 2009).

RKY is a TCM formula composed of several specific Chinese medicines for treating breast cancer (**Figure 2**). One study pretreated with RKY found that RKY might inhibit the lymph node metastasis of breast cancer mainly through decreasing the expressions of VEGF-C and VEGFR-3 and inhibit lymphangiogenesis (Li and Dang, 2011). Another study from the same team has shown that RKY inhibited lymphangiogenesis partly by interfering with the Ang-2/Tie2 signaling pathway—inhibiting the protein expression of Ang-2 (Sun and Li, 2013). This indicates that RKY can inhibit lymphangiogenesis by affecting different signaling pathways. Importantly, Treatment with TCM formula can greatly make use of the characteristics of its multiple drug components and the advantages of multi-target therapy.

JFK, an oral liquid of Chinese herbal, has been used to treat NSCLC in clinics (**Figure 2**). The current research suggests that JFK prevents the migration and differentiation of LECs *via* regulating the expression of VEGF-C/VEGFR-3 and the SDF-1/CXCR4. Consequently, JFK can suppress NSCLC through antilymphangiogenesis and is a promising antilymphangiogenesis agent (He et al., 2016). Another *in vivo* experimental study shows that bone marrow–derived mesenchymal stem cells (BMMSCs) differentiate into LECs by metastasis to tumors and participate in lymphangiogenesis of lung cancer in mice. Significantly, JFK inhibits the growth of lung tumors *via* suppressing the transformation of BMMSCs and lymphangiogenesis (Zhou et al., 2017).

JG-DS, composed of *Pinellia*, Celestial Star, *Poria*, white mustard, Chen Pi, Full Scorpion, Chicken inside gold, and Licorice, is an own Chinese medicine granules for inhibiting the recurrence and metastasis of cancer. Feng et al. found that JG-DS inhibited the tube formation and metastasis of human LECs *in vitro*, thereby directly inhibiting lymphangiogenesis (Feng et al., 2016).

PZH, the main active ingredients include *Moschus*, Calculus Bovis, Snake Gall, and Radix Notoginseng, is a well-known traditional Chinese formula (**Figure 2**). Using different human colorectal cancer cell lines and human LECs model stimulated by VEGF-C, Lin et al. evaluated the effects of PZH on tumor metastasis and VEGF-C expression. It was found that PZH suppresses lymphangiogenesis *via* the downregulation of VEGF-C, which may be a potential molecular mechanism of inhibition of metastasis of colorectal cancer by PZH (Lin et al., 2016).

### Clinical Research of TCM Formula

It is worth mentioning that related clinical studies show that two TCM formulas, Shu-GanJianPi formula and TaoHongSiWu formula, are inhibitors of lymphangiogenesis (**Figure 2**). In the first clinical study, all breast cancer patients (50 people) received CTF chemotherapy, and the test group (30 people) treated with Shugan Jianpi formula at the same time. After two courses of chemotherapy, all patients were operated on, and the MLVD and the expression of Ang-2 and nm23 in breast cancer tissues were detected by Bard biopsy needle biopsy, histology, and immunohistochemistry. The results show that ShuGanJianPi formula inhibits lymphatic metastasis in breast cancer by interfering with the expression of Ang-2 and nm23, which affects lymphangiogenesis (Jin and Li, 2012). Another clinical trial found that TaoHongSiWu Decoction can inhibit tumor lymphangiogenesis in breast cancer patients. Its mechanism of action is related to the inhibition of VEGF-C and LVD expression (Chu et al., 2013).

Taken together, there is good reason to believe that TCM regulating lymphangiogenesis may become a new tumor treatment method.

## Conclusion and Perspectives

In the past 20 years, clinical and experimental studies have revealed the basic role of lymphatics in the pathogenesis of many different diseases and revealed the potential role of lymphangiogenesis regulation in treatment and intervention. In fact, experimental studies have fully demonstrated the principle that although inhibition of lymphangiogenesis can limit tumor metastasis and potential graft rejection, stimulation of lymphangiogenesis can accelerate the regression of inflammation and edema. At present, most of the treatment studies focus on the VEGFC–VEGFD–VEGFR3 pathway, including most of extracts from TCM the above mentioned, Injections of single TCM and formula.

In addition to VEGFC-VEGFD-VEGFR3 pathway, some of these TCM may work through an different mechanism. For example, licoricidin, GSPP/Gecko, curcumin, *Astragalus* injection, cinobufacini injection, JFK can inhibit lymphangiogenesis by suppressing the expression of LYVE-1, bFGF, inhibiting HMGB1/VEGF-D signaling pathway, reducing the positive rate of HIF-1α, attenuating the expression of HGF, and weakening the expression of the SDF-1/CXCR4, respectively.

Moreover, PNS, promoted lymphangiogenesis, may be an attractive and suitable drug for the treatment of secondary lymphedema or other diseases related to lymphadenectomy. The combination of TCM and lymphangiogenesis provides an additional effect in the study of the regulation of lymphangiogenesis and related diseases.

The regulatory effect of TCM on the lymphatic system has been confirmed in a variety of *in vivo* and *in vitro* models and some clinical experiments. In the past few years, advances in molecular extraction and synthesis technology of TCM, imaging technology, and animal models have helped us witness the important role of TCM in regulating lymphangiogenesis in many diseases, including cardiovascular disease, inflammation, and wound healing, especially cancer. Lymphangiogenesis is the main cause of the development and change of many diseases, and it even directly affects the pathological progress of disease. Currently, there are no agents specifically interfere with lymphangiogenesis, so it is urgent to develop effective agents to interfere with lymphangiogenesis. Now, the research on lymphangiogenesis regulators and the targets of TCM for regulating lymphangiogenesis has captured more attention. These research advances will provide new insights into the role of TCM in regulating lymphangiogenesis. Importantly, anti-lymphangiogenesis might become a new disease treatment method.

## Author Contributions

LP, YhW, and CZha conceived and designed the study. LP and YhW performed the experiments. LP and YhW wrote and revised the paper. YD, HF, MC, QW, YW, CZho, SL, and CZha reviewed and edited the manuscript. All authors contributed to the article and approved the submitted version.

## Funding

This research was funded by National Natural Science Foundation of China (no. 81873264), Technological Innovation Action Plan of the Science and Technology Commission of Shanghai Municipality (no.15401902500 and no.17401901600) and Longhua Medical Scholar Project (no. LYTD-86).

## Conflict of Interest

The authors declare that the research was conducted in the absence of any commercial or financial relationships that could be construed as a potential conflict of interest.

## Abbreviations

TCM, Traditional Chinese Medicine; ART, artemisinin; DHA, dihydroartemisinin; GS-Rg3, 20(R)-ginsenoside Rg3; GS-Rh2, ginsenoside Rh2; CPT, cryptotanshinone; GS-Rg1, Ginsenoside Rg1; HIF-1, Hypoxia-inducible factor-1; Ang-1/2, Angopoietin-1/2; MMP-9, matrix metalloproteinase-9; VEGF, vascular endothelial growth factor; LECs, lymphatic endothelial cells; LY, lymphangiogenesis; MOA, mechanism of action; FGF-2, fibroblast growth factor-2; IGF-1/2, Insulin-like growth factor-1/2; PDGF-BB, platelet derived growth factor-BB; HGF, hepatocyte growth factor; TNF, tumor necrosis factor; IL, interleukin; CLEC14A, C-type lectin family 14 member A; ET-1, Endothelin-1; TSP-1, Thrombospondin-1; TGF-β1, Transforming Growth Factor; IFN-γ, Interferon; BMP2, Bone morphogenetic protein; NCTD, Norcantharidin; PNS, Total saponins of panaxnotoginseng; DHJST, DuHuoJiShengTang; FJHQ, FangjiHuangqi; VRIN, VEGFR3-mediated immune-nanoemulsion of ginsenoside Rg3; GSPP, Gekko Sulfated Glycopeptide; HDLEC, human dermal lymphatic endothelial cells; MLVD, micro lymphatic vessel density; RKY, RuKangYin; JFK, JinFuKang; NSCLC, non-small cell lung cancer; JG-DS, Jinlongshe Granule drug-containing serum; LVD, lymphatic vessel density; HDW, Hedyotis diffusa Willd; EEHDW, Ethanol extract of HDW; PZH, Pien Tze Huang.
